# High Levels of *Helicobacter pylori* Antimicrobial Resistance in Ireland—A Multicentre Study

**DOI:** 10.3390/microorganisms14030704

**Published:** 2026-03-21

**Authors:** Thomas J. Butler, Stephen Molloy, Atiyekeogbebe Douglas, Denise Brennan, Rebecca FitzGerald, Conor Costigan, Vikrant Parihar, Kevin Van Der Merwe, Serhiy Semenov, Donal Tighe, Sharon Hough, David Kevans, Colm O’Morain, Deirdre McNamara, Sinéad Marian Smith

**Affiliations:** 1 School of Medicine, Trinity College Dublin, D02 PN40 Dublin, Ireland; thbutler@tcd.ie (T.J.B.); stmolloy@tcd.ie (S.M.); douglaar@tcd.ie (A.D.); dbrenna9@tcd.ie (D.B.); fitzger4@tcd.ie (R.F.); cocostig@tcd.ie (C.C.); kevansd@tcd.ie (D.K.); colmomorain@gmail.com (C.O.); mcnamad@tcd.ie (D.M.); 2 Department of Gastroenterology, Tallaght University Hospital, D24 NR0A Dublin, Ireland; 3 Department of Gastroenterology, Letterkenny University Hospital, Letterkenny, F92 AE81 Donegal, Ireland; vikpar37@yahoo.com (V.P.); kvandm91@gmail.com (K.V.D.M.); 4 Department of Gastroenterology, Mayo University Hospital, Castlebar, F23 H529 Mayo, Ireland; semenov@hse.ie (S.S.); donal.tighe1@hse.ie (D.T.); 5 Department of Gastroenterology, St. James’s Hospital, D08 NHY1 Dublin, Ireland; shough@stjames.ie

**Keywords:** *Helicobacter pylori*, antimicrobial resistance, antimicrobial susceptibility testing, clarithromycin, metronidazole, levofloxacin

## Abstract

Resistance surveillance programmes are essential for choosing the most appropriate eradication therapy for the stomach pathogen *Helicobacter pylori*. This study aimed to determine *H. pylori* antimicrobial resistance rates in Ireland. *H. pylori* was cultured from patients attending four gastroenterology clinics from 2018 to 2023. Antimicrobial susceptibility testing (AST) was performed using Etests for metronidazole, clarithromycin, levofloxacin, amoxicillin, tetracycline and rifampicin and resistance classified using EUCAST guidelines. Resistance rates were compared between *H. pylori* treatment-naïve and previously treated patients (primary and secondary resistance, respectively). Samples from 138 culture-positive patients (mean age 49.4 ± 15.7 years, 47.1% female) were analysed. A total of 28.7% of isolates from treatment-naïve patients were susceptible to all antimicrobials tested. Primary resistance rates to metronidazole, clarithromycin, levofloxacin, amoxicillin, tetracycline and rifampicin were 44.3%, 36.5%, 18.3%, 14.6%, 9.6% and 9.6%, respectively. Primary dual resistance to clarithromycin and metronidazole was 22.6% and primary multidrug resistance was 13.0%. Secondary resistance rates were significantly higher than primary resistance rates for clarithromycin, metronidazole, dual resistance to clarithromycin and either amoxicillin, metronidazole or levofloxacin, and multidrug resistance. Female sex and older age were associated with increased risk of resistance. *H. pylori* resistance rates were high in our cohort. Clarithromycin-based triple therapy should no longer be used in Ireland in the absence of pre-treatment AST. Resistance to amoxicillin, tetracycline and rifampicin should be monitored closely.

## 1. Introduction

*Helicobacter pylori* (*H. pylori*) is a Gram-negative, microaerophilic, spiral-shaped, bacterium [1,2,3,4] present in the stomach of almost half of the population worldwide [5,6]. Infection causes chronic gastritis and increases the risk of developing peptic ulcer disease. Additionally, *H. pylori* is the main etiological agent in gastric cancer, which is the fifth most common malignancy globally and the fifth leading cause of cancer-related deaths [3,5,7,8,9,10,11]. *H. pylori* has been designated a class I (definite) carcinogen by the World Health Organization (WHO) since 1994 [12]. Infection is usually acquired during childhood and persists for life in the absence of eradication therapy. The bacteria produce a variety of factors that facilitate colonisation of the gastric mucosa, including the urease enzyme, which protects against the low pH of the stomach, flagella that enable motility and several outer membrane proteins involved in attachment [2,3,4]. *H. pylori* is detected using non-invasive diagnostic tests or invasively using samples obtained during gastroscopy. Non-invasive test options include the urea breath test, stool antigen test, or serology, while the most commonly used invasive methods are the rapid urease test, histology, bacterial culture and PCR [3,4,13].

Treatment involves a combination of antimicrobials and an acid-suppressing drug. First-line therapy has traditionally comprised a proton pump inhibitor (PPI), clarithromycin and amoxicillin. Amoxicillin is replaced with metronidazole in penicillin-allergic patients [3,4]. However, these triple therapies have become less effective over time, mainly due to the emergence of antimicrobial-resistant *H. pylori*. As a result, international consensus guidelines now recommend that first-line therapy is guided by the local prevalence of *H. pylori* antimicrobial resistance. Empirical clarithromycin-based triple therapy should not be used in areas where primary clarithromycin resistance is >15%, with bismuth quadruple therapy (PPI, bismuth salt, metronidazole and tetracycline), non-bismuth concomitant therapy (PPI, bismuth, metronidazole, clarithromycin) or high-dose PPI–amoxicillin dual therapy as alternative options [14,15,16]. Second-line treatment and rescue therapies should never be the same as the previously prescribed anti-*H. pylori* therapies. Levofloxacin triple or quadruple therapies are second-line options, and rifabutin triple therapy is an option for rescue therapy [14,15,16].

It is well established that *H. pylori* resistance rates vary across geographic regions [17,18,19,20,21,22,23,24]. As treatment recommendations are based on the regional prevalence of antimicrobial resistance, local resistance surveillance programmes are important for choosing the most appropriate eradication therapy in each population. Thus, the aim of this study was to determine *H. pylori* antimicrobial resistance rates in Ireland. Secondly, we investigated patient risk factors associated with harbouring antimicrobial-resistant *H. pylori*.

## 2. Materials and Methods

### 2.1. Ethical Approval

The study was approved by the Joint Research Ethics Committee of Tallaght University Hospital and St. James’s Hospital (Reference: REC-2013/23/04/2014-11-List 41(11) and REC-2020-03-List 9—Amendment (18)), the Research Ethics Committee of Letterkenny University Hospital (Reference: *Helicobacter pylori* antibiotic resistance) and the Research Ethics Committee of Mayo University Hospital (Reference: ToM/MV 20220201). The procedures followed were in accordance with the ethical standards of the Helsinki Declaration of 1975, as revised in 2000.

### 2.2. Study Population and Sample Collection

Patients attending for upper gastrointestinal endoscopy at Tallaght University Hospital and St. James’s Hospital, Co. Dublin, Letterkenny University Hospital, Co. Donegal and Mayo University Hospital, Co. Mayo, between January 2018 and June 2023 were invited to participate in the study. Inclusion criteria were (1) ability and willingness to participate in the study and to provide informed consent; and (2) confirmed *H. pylori* infection by culture. Exclusion criteria were (1) age less than 18 years; (2) pregnancy or lactation; (3) severe intercurrent illness; (4) recent antimicrobial use (within 4 weeks); and (5) bleeding problems or use of blood thinning drugs. Upon receipt of informed consent, one corpus and one antrum biopsy were taken from each patient and placed directly into sterile DENT’s transport medium (brain heart infusion broth containing 2.5% (*w*/*v*) yeast extract, 5% (*v*/*v*) horse serum and *H. pylori* Selective Supplement (Oxoid, (Hampshire, UK)) and transported to the Meath Foundation Research Laboratory, Trinity Centre, Tallaght University Hospital, for culture and antimicrobial susceptibility testing. Patient demographics and *H. pylori* treatment history were recorded.

### 2.3. Culture of H. pylori

Using a sterile inoculating loop, the corpus and antrum biopsies from each patient were removed from the transport medium and spread directly onto a Columbia blood agar plate containing 5% (*v*/*v*) laked horse blood. The biopsies were removed from the agar and the plates incubated at 37 °C under microaerobic conditions using the CampyGen system (Oxoid) for up to 7 days. *H. pylori* was identified by visual inspection of the colonies, a positive urease test and by PCR.

### 2.4. Antimicrobial Susceptibility Testing (AST)

Using a sterile cotton swab, *H. pylori* colonies were dispersed into maximum recovery diluent (Oxoid) until the turbidity was that of a 3 McFarland standard. The inoculum from each cultured strain was spread evenly onto 6 Columbia blood agar plates containing 5% (*v*/*v*) laked horse blood. One Etest strip (Biomerieux, Craponne, France) for clarithromycin, metronidazole, amoxicillin, levofloxacin, tetracycline or rifampicin was applied to each inoculated plate using a sterile forceps. Rifabutin Etests are not available, and therefore rifampicin was used to determine sensitivity to this class of antimicrobials. The plates were incubated at 37 °C for 48–72 h under microaerobic conditions. AST results were interpreted according to the guidelines and criteria of the European Committee on Antimicrobial Susceptibility Testing (EUCAST, Clinical Breakpoint Tables Version 13.4, valid from 29 June 2023). The following minimum inhibitory concentration (MIC) cut-offs for resistance were used: clarithromycin: >0.25 mg/L; metronidazole: >8 mg/L, amoxicillin: >0.125 mg/L, levofloxacin: 1 mg/L, tetracycline: >1 mg/L; and rifampicin >1 mg/L. Quality control AST experiments were performed using the strain CCUG 18742, which is susceptible to all antimicrobials tested.

### 2.5. Statistical Analysis

All statistical analyses were performed using GraphPad Prism, version 9.5.1 (GraphPad Software, San Diego, CA, USA). Continuous variables are expressed as the mean ± standard deviation (SD), while categorical variables are presented as frequencies and percentages. Resistance rates were compared between *H. pylori* treatment-naïve and previously treated patients (primary and secondary resistance, respectively). Differences between categorical variables were analysed using the two tailed Chi-square test.

To evaluate the relationship between patient age and antibiotic resistance, binary logistic regression analysis was performed, with age entered as a continuous independent variable and antimicrobial susceptibility result (resistant or susceptible) as the dependent variable. Regression coefficients (β_0_ for intercept and β_1_ for slope) were obtained for each antibiotic and for combinations of dual or multidrug resistance (resistant to 3 or more antimicrobials). Model fit was assessed using the likelihood ratio test (LRT), and statistical significance of the regression model was determined based on the *p* value of the LRT. For all statistical analysis, a *p* value of <0.05 was considered significant.

## 3. Results

### 3.1. Patient Demographics

Samples from 138 culture-positive patients were included in the study. The mean age of the study population was 49.4 ± 15.7 years and 47.1% (*n* = 65) were female (Table 1). A total of 83.3% (*n* = 115) of the study population was treatment-naïve for *H. pylori*, while 16.7% (*n* = 23) had previously undergone treatment for the infection (Table 1). There was no significant difference in age or sex between the treatment-naïve and previously treated groups (Table 1).

### 3.2. Antimicrobial Resistance Rates

**Figure 1:**
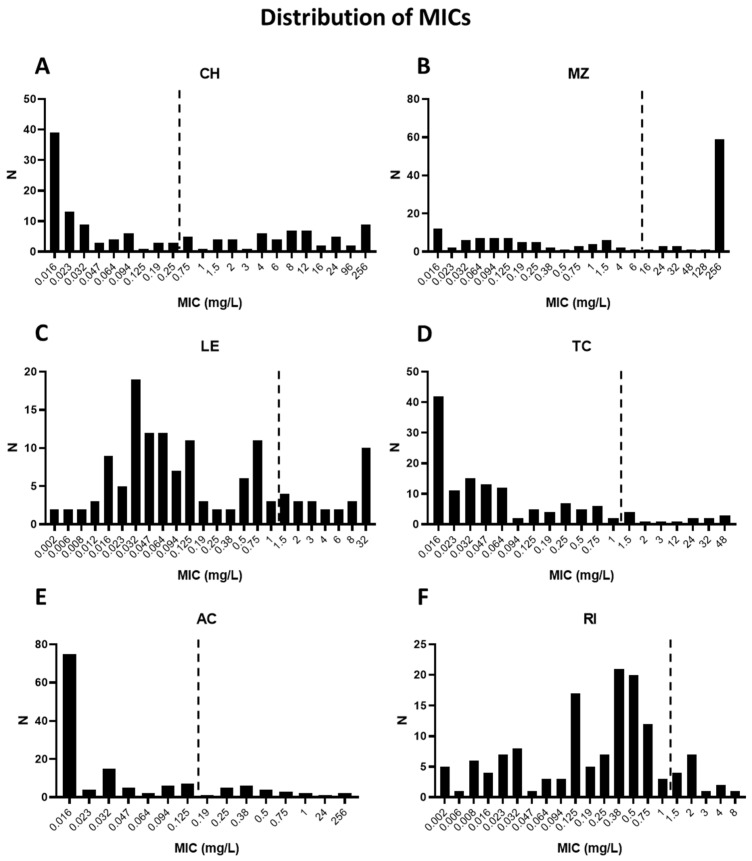
Distribution of minimum inhibitory concentration (MIC) values for each antimicrobial across the clinical isolates tested. (**A**) CH: clarithromycin. (**B**) MZ: metronidazole. (**C**) LE: levofloxacin. (**D**) TC: tetracycline. (**E**) AC: amoxicillin. (**F**) RI: rifampicin. N: number of strains. The dashed lines represent the EUCAST MIC breakpoints for resistance.

The distributions of MICs obtained across the cultured isolates for each antimicrobial are shown in Figure 1. Overall resistance rates to clarithromycin and metronidazole were high at 41.3% (*n* = 57/138) and 49.3% (*n* = 68/138), respectively (Table 2). Overall resistance rates to levofloxacin, tetracycline, amoxicillin and rifampicin were 19.6% (*n* = 27/138), 10.1% (*n* = 14/138), 17.4% (*n* = 24/138) and 10.9% (*n* = 15/138), respectively.

Of the treatment-naïve group, only 28.7% (*n* = 33/115) of isolates tested were susceptible to all six of the antibiotics tested. A significant decrease in the susceptibility profile was noted in isolates from those previously treated, with only one isolate (4.3%) susceptible to all six antimicrobials (*p* = 0.01; Table 2). Primary resistance rates to clarithromycin and metronidazole in isolates from treatment-naïve patients were high at 36.5% (*n* = 42/115) and 44.3% (*n* = 51/115), respectively, with dual resistance to both clarithromycin and metronidazole observed in 22.6% (*n* = 26/115) of isolates. A total of 13.0% (*n* = 15/115) of isolates was multidrug resistant (Table 2).

When rates were compared between treatment-naïve isolates and those from previously treated individuals, the resistance to clarithromycin significantly increased from 36.5% (*n* = 42/115) to 65.2% (*n* = 15/23) (*p* = 0.01) and resistance to metronidazole increased from 44.3% (*n* = 51/115) to 73.9% (*n* = 17/23) (*p* = 0.01; Table 2). There was also a significant increase in dual resistance to clarithromycin and amoxicillin (8.7% (*n* = 10/115) to 26.1% (*n* = 6/23); *p* = 0.02), clarithromycin and metronidazole (22.6% (*n* = 26/115) to 52.2% (*n* = 12/23); *p* < 0.005) and clarithromycin and levofloxacin (7.8% (*n* = 9/115) to 21.7% (*n* = 5/23); *p* = 0.04) when rates were compared between samples from treatment-naïve versus previously treated patients (Table 2). Multidrug resistance increased from 13.0% (*n* = 15/115) in treatment-naïve isolates to 43.5% (*n* = 10/23) (*p* < 0.001) in isolates from previously treated patients (Table 2).

### 3.3. Factors Associated with Antimicrobial Resistance

Next, factors associated with antimicrobial resistance were investigated. Only 13.8% (*n* = 9/65) of females were infected with isolates susceptible to all six antimicrobials, compared to 34.2% (*n* = 25/73) of males (*p* < 0.01; Table 3). In line with this, overall resistance was significantly higher in isolates from females than males with regard to clarithromycin (56.9% (*n* = 37/65) versus 27.4% (*n* = 20/73), respectively; *p* < 0.001) and amoxicillin (24.6% (*n* = 16/65) versus 11% (*n* = 8/73), respectively; *p* = 0.03). Dual resistance to both clarithromycin and amoxicillin was also higher in females than males (20% (*n* = 13/65) versus 4.1% (*n* = 3/73), respectively; *p* < 0.005), as was dual resistance to both clarithromycin and metronidazole (43.1% (*n* = 28/65) versus 13.7% (*n* = 10/73), respectively; *p* < 0.001) (Table 3).

Overall resistance rates were also compared among isolates from younger compared to older patients, using the WHO definition of older age as 60 years and above [25]. A total of 24.6% (*n* = 34/138) of patients from whom *H. pylori* was cultured were ≥60 years, with a mean age of 71.3 ± 6.9 years compared to 42.3 ± 10.2 years (*p* < 0.0001) in the under 60-year-old group (Table 4). Levofloxacin resistance was more than twice as high in isolates from older compared to younger patients (38.2% (*n* = 13/34) versus 13.5% (*n* = 14/104), respectively, *p* < 0.005; (Table 5)). In line with this finding, dual resistance to levofloxacin and either clarithromycin or metronidazole was significantly higher in *H. pylori* isolates from ≥60 years compared to <60 years (Table 5). Multidrug resistance was also more prevalent in *H. pylori* isolates from the older compared to younger patients (32.4% (*n* = 11/34) vs. 13.5% (*n* = 14/104), respectively, *p* < 0.01; (Table 5)). Finally, logistic regression analysis also showed that increasing age was a risk factor for levofloxacin resistance, dual levofloxacin resistance and dual levofloxacin and metronidazole resistance (Table 6).

## 4. Discussion

In this study, we evaluated resistance to six antimicrobials by culturing *H. pylori* from patients attending four gastroenterology clinics on the island of Ireland between 2018 and 2023. We used a combined corpus and antrum biopsy sampling approach, as combined biopsy sampling improves *H. pylori* culture success compared to single antrum biopsy sampling [26,27,28], takes into account the patchy distribution of *H. pylori* in the stomach, which can occur from using PPIs [27,29,30], and also captures any differences in the antimicrobial susceptibility profiles between isolates from the corpus and antrum of the same patient [31,32].

*H. pylori* antimicrobial resistance rates were high in isolates from our patient cohort and have increased over time. Primary clarithromycin resistance has risen from 3.9% in 1997 to 9.3% in 2007/8 [33], to 36.5% in the current study, while primary metronidazole resistance has increased from 27.1% and 29.1% in 1997 and 2007/8, respectively [33], to 44.3% in the current study. Primary *H. pylori* resistance to levofloxacin in Ireland has risen from 11.2% in 2008/9 [34,35] to 18.3% in the current study. Our primary clarithromycin resistance rate is higher than the pooled primary clarithromycin resistance rates reported in pan-European studies, which range from 21 to 25% [17,36,37], but similar to Greece, Croatia and Italy, where primary clarithromycin resistance is also above 30% [17]. Our primary metronidazole and levofloxacin resistance rates are similar to the pooled pan-European resistance rates (27–39% and 15.8–20%, respectively) [17,36,37] and other countries worldwide [18,19]. Higher primary resistance to amoxicillin, rifampicin and tetracycline (14.8%, 9.6% and 9.6%, respectively) was observed among the *H. pylori* isolates collected in this study compared to other regions, particularly in Europe. The detection of primary amoxicillin resistance herein is reflected by the poor eradication rate recently obtained using first-line high-dose amoxicillin and PPI dual therapy in a recent multicentre study in our population [38].

Although resistance to amoxicillin, tetracycline and rifampicin has been considered uncommon in the past, increasing studies have reported emerging resistance to these antimicrobials. Evidence from Europe shows an emergence of amoxicillin resistance in the Netherlands and the Eastern Mediterranean region, with resistance rates of 10% [39] and 14% [18], respectively. A systematic review and meta-analysis of primary antibiotic resistance in the Asia-Pacific region revealed amoxicillin resistance rates of 8%, 10%, 18% and 30% in isolates from Saudia Arabia, Iran, India and Pakistan, respectively [23]. Very high primary amoxicillin resistance has been reported in some African countries, with rates of 34%, 82% and 100% in the Democratic Republic of Congo [40], Egypt [41], and Cameroon [42], respectively. With regard to primary tetracycline resistance, rates of 5% and 10% have been described in Poland [43] and the Eastern Mediterranean [18], respectively. Similarly, primary tetracycline resistance rates between 6% and 12% have been described in the Asia-Pacific region [23] and between 34% and 38% in some African countries [40,41]. Finally, primary resistance to rifampicin has also been shown in Lithuania (8%, [44]), Latvia (10%, [45]), Spain (33%, [46]), and Egypt (63%, [41]).

The primary resistance rates observed here, particularly in relation to amoxicillin, tetracycline and rifampicin, provide a rationale for continued surveillance and investigation. Indeed, experiments are currently ongoing to determine the molecular mechanisms of resistance in our isolates. Variations between our resistance rates and those from other countries are also worth considering. There is a strong link between primary *H. pylori* resistance and previous antimicrobial use [17,34,47,48]. Analysis of antimicrobial consumption in Europe has shown a significant association between the consumption of macrolides in the community and *H. pylori* clarithromycin resistance, and between the consumption of quinolones in the community and levofloxacin resistance [17,34]. Studies from the UK and USA have also shown that previous antimicrobial use increases the risk of harbouring resistant strains of *H. pylori* [47,48]. Therefore, variations in antimicrobial prescribing patterns are likely to influence the emergence of *H. pylori* resistance in different regions. Unfortunately, a full history of antimicrobial use among the individual patients recruited in this study was unavailable. However, it is noteworthy that Ireland has a proportionately higher use of antimicrobials compared to many other countries in the EU and that the consumption of β-lactam antibiotics and tetracyclines has been steadily increasing over the years [49].

Among the isolates collected from *H. pylori* treatment-naïve patients, only 28.7% were susceptible to all six of the antimicrobials tested. A significantly lower percentage (4.3%) of isolates obtained from those previously treated for *H. pylori* infection was susceptible to all six antimicrobials. In line with this observation, resistance to clarithromycin was significantly higher in isolates from previously treated patients compared to those from treatment-naïve individuals (65.2% vs. 36.5%, respectively). This finding is not surprising given that clarithromycin-based triple therapy was shown to be the most common first-line treatment prescribed for *H. pylori* in Ireland between 2013 and 2022, accounting for 88% of prescribed regimens analysed [50]. Secondary metronidazole resistance was also higher compared to primary metronidazole resistance (73.9% vs. 44.3%, respectively), as was dual resistance to clarithromycin and amoxicillin (26.1% vs. 8.7%, respectively), dual resistance to clarithromycin and metronidazole (52.2% vs. 22.6%, respectively) and dual resistance to clarithromycin and levofloxacin (21.7% vs. 7.8%, respectively) when rates were compared between samples from previously treated patients versus treatment-naive. Multidrug resistance increased from 13% in treatment-naïve isolates to 43.5% in isolates from previously treated patients. These findings of increased resistance among isolates from patients previously treated for *H. pylori* highlight the challenges associated with second-line and rescue therapy and the importance of selecting the most appropriate *H. pylori* eradication strategy for first-line treatment.

Female sex and older age were associated with a higher risk of harbouring antimicrobial-resistant *H. pylori*. Only 13.8% of females were infected with isolates susceptible to all six antimicrobials, compared to 34.2% of males, and overall resistance was significantly higher in isolates from females than males with regard to clarithromycin (56.9% vs. 27.4%, respectively), amoxicillin (24.6% vs. 11.0%, respectively) and dual resistance to both clarithromycin and amoxicillin (20% vs. 4.1%, respectively) or clarithromycin and metronidazole (43.1% vs. 13.7%, respectively). Levofloxacin resistance was more than twice as high in isolates from older compared to younger patients (38.2% vs. 13.5%, respectively), while dual resistance to levofloxacin and either clarithromycin or metronidazole and multidrug resistance were also significantly higher in *H. pylori* isolates from ≥60 years compared to <60 years. Logistic regression analysis also revealed that increasing age was a risk factor for levofloxacin resistance, dual levofloxacin resistance and dual levofloxacin and metronidazole resistance. Both older age and female sex have previously been reported as risk factors for *H. pylori* antimicrobial resistance [18,33,34,51,52]. Indeed, previous studies from Ireland [33], Europe [34,51,52,53,54,55,56,57] and other parts of the world [58,59,60,61,62,63,64] have shown that *H. pylori* antimicrobial resistance is more likely to occur in isolates from females compared to males. These findings are likely to be influenced by previous antibiotic use. Older patients are likely to have been exposed to more courses of antimicrobials than younger patients. Women may also be more likely to have increased antimicrobial exposure compared to men, as they have been reported to seek primary care consultation more frequently [65]. Higher metronidazole resistance in isolates from females may be due to the use of metronidazole for gynaecological infections, such as bacterial vaginosis and trichomoniasis [52]. DNA mutations (A2142G and A2143G) in the 23S rRNA gene associated with clarithromycin resistance in *H. pylori* confer cross-resistance to other antimicrobials, such as clindamycin [66]. Such overlapping resistance mechanisms suggest that higher clarithromycin resistance in *H. pylori* from females may also result from the treatment of gynaecological infections, as clindamycin is used as an alternative to metronidazole for bacterial vaginosis [52]. It is possible that other factors, such as differences in bacterial load, physiology, hormones and/or immune responses to *H. pylori*, may play a role in the development of resistance in females compared to males and these areas are worthy of further study. In line with higher resistance rates in *H. pylori* isolated from women, studies have shown that female sex is a risk factor for treatment failure, with lower eradication rates reported in women compared to men [67,68,69].

The strengths of our study include the combined corpus and antrum biopsy sampling approach to culture *H. pylori* and the use of standardised antimicrobial susceptibility testing methods recommend by the European Helicobacter and Microbiota Study Group [17,34]. Further, this is the first resistance study carried out in Ireland that investigated resistance to all six antimicrobials in clinical use for *H. pylori* and includes samples from more than one hospital site. Information was collected on *H. pylori* treatment history, which enabled analysis of primary and secondary resistance rates. However, comprehensive data on total previous antibiotic use for individual patients were not available. Data on the treatment prescribed to the patients included in the study and the treatment outcomes were also unavailable, preventing direct analysis of the risk of harbouring a resistant strain and treatment failure in our cohort.

In summary, high rates of *H. pylori* antimicrobial resistance were observed in clinical isolates from patients in Ireland. The high rate of primary clarithromycin has important implications for *H. pylori* treatment. Primary clarithromycin resistance decreases the efficacy of the standard clarithromycin–amoxicillin–PPI triple therapy by 70% [34,70,71] and should not be used in first-line therapy in areas where primary clarithromycin resistance is >15% [14]. Indeed, based in part on the data presented herein, updated Irish consensus guidelines now recommend that clarithromycin triple therapy should only be used in cases where clarithromycin susceptibility has been confirmed [72]. Non-bismuth concomitant therapy (PPI, clarithromycin, metronidazole, amoxicillin) is not a suitable first-line alternative in our population due to the high dual clarithromycin and metronidazole resistance rate. Bismuth quadruple therapy (PPI, bismuth salt, metronidazole and tetracycline) is now the recommended first-line treatment [72] and is known to overcome metronidazole resistance [14,73]. In the absence of AST, knowledge of a patient’s antibiotic history may prove useful in therapy decision making, especially in females and older patients. Finally, emerging resistance to amoxicillin, tetracycline and rifampicin and the associated impacts on treatment outcomes are worth monitoring closely.

## 5. Conclusions

Resistance rates were high in *H. pylori* isolated from patients in Ireland. Female sex and older age were associated with increased risk of resistance. Clarithromycin-based triple therapy should no longer be used as first-line treatment in Ireland in the absence of pre-treatment AST. Resistance surveillance should be continued, especially in light of emerging resistance to amoxicillin, tetracycline and rifampicin.

## Figures and Tables

**Table 1:** Demographics of included patients.

	Overall *n* = 138	Treatment-Naïve *n* = 115	Previously Treated *n* = 23	*p* Value ^1^
Mean age (years)	49.4	49.6	48.8	0.83
SD	15.7	16.3	12.2	
Sex n (%)				0.15
Male	73 (52.9%)	64 (55.7%)	9 (39.1%)	
Female	65 (47.1%)	51 (44.3%)	14 (60.9%)	

^1^ Treatment-naïve versus previously treated; SD: standard deviation.

**Table 2:** Rates of resistance to each antimicrobial.

	Overall *n* = 138	Treatment-Naïve *n* = 115	Previously Treated *n* = 23	*p* Value ^1^
No Resistance	34 (24.7%)	33 (28.7%)	1 (4.3%)	0.01 *
Clarithromycin	57 (41.3%)	42 (36.5%)	15 (65.2%)	0.01 *
Metronidazole	68 (49.3%)	51 (44.3%)	17 (73.9%)	0.01 *
Levofloxacin	27 (19.6%)	21 (18.3%)	6 (26.1%)	0.39
Tetracycline	14 (10.1%)	11 (9.6%)	3 (13.0%)	0.61
Amoxicillin	24 (17.4%)	17 (14.8%)	7 (30.4%)	0.07
Rifampicin	15 (10.9%)	11 (9.6%)	4 (17.4%)	0.27
Dual C + A	16 (11.6%)	10 (8.7%)	6 (26.1%)	0.02 *
Dual C + M	38 (27.5%)	26 (22.6%)	12 (52.2%)	<0.005 *
Dual C + L	14 (10.1%)	9 (7.8%)	5 (21.7%)	0.04 *
Dual M + L	20 (14.5%)	14 (12.2%)	6 (26.1%)	0.08
Multi (3+)	25 (18.1%)	15 (13.0%)	10 (43.5%)	<0.001 *

^1^ Isolates from treatment-naïve patients versus those from previously treated patients; * statistically significant; C: clarithromycin; A: amoxicillin; M: metronidazole; L: levofloxacin; Multi (3+): resistance to 3 or more antimicrobials.

**Table 3:** Antimicrobial resistance rates according to patient sex.

	Male *n* = 73	Female *n* = 65	*p* Value
No Resistance	25 (34.2%)	9 (13.8%)	<0.01 *
Clarithromycin	20 (27.4%)	37 (56.9%)	<0.001 *
Metronidazole	32 (43.8%)	36 (55.4%)	0.18
Levofloxacin	15 (20.5%)	12 (18.5%)	0.76
Tetracycline	5 (6.8%)	9 (13.8%)	0.17
Amoxicillin	8 (11.0%)	16 (24.6%)	0.03 *
Rifampicin	8 (11.0%)	7 (10.8%)	0.97
Dual C + A	3 (4.1%)	13 (20.0%)	<0.005 *
Dual C + M	10 (13.7%)	28 (43.1%)	<0.001 *
Dual C + L	6 (8.2%)	8 (12.3%)	0.43
Dual M + L	11 (15.1%)	9 (13.8%)	0.84
Multi (3+)	9 (12.3%)	16 (24.6%)	0.06

* Statistically significant; C: clarithromycin; A: amoxicillin; M: metronidazole; L: levofloxacin; Multi (3+): resistance to 3 or more antimicrobials.

**Table 4:** Demographics of those < 60 years versus ≥60 years.

	<60 Years *n* = 104	≥60 Years *n* = 34	*p* Value ^1^
Mean age (years)	42.3	71.3	<0.0001 *
SD	10.2	6.9	
Sex N (%)			0.05
Male	50 (48.1%)	23 (67.7%)	
Female	54 (51.9%)	11 (32.4%)	

SD: standard deviation; ^1^ <60 years versus ≥60 years; * statistically significant.

**Table 5:** Antimicrobial resistance rates in those < 60 years versus ≥60 years.

	<60 Years *n* = 104	≥60 Years *n* = 34	*p* Value ^1^
No Resistance	23 (22.1%)	11 (32.4%)	0.23
Clarithromycin	44 (42.3%)	13 (38.2%)	0.68
Metronidazole	50 (48.1%)	18 (52.9%)	0.62
Levofloxacin	14 (13.5%)	13 (38.2%)	<0.005 *
Tetracycline	10 (9.6%)	4 (11.8%)	0.72
Amoxicillin	16 (15.4%)	8 (23.5%)	0.28
Rifampicin	13 (12.5%)	2 (5.9%)	0.28
Dual C + M	26 (25.0%)	12 (35.3%)	0.24
Dual C + A	11 (10.6%)	5 (14.7%)	0.51
Dual C + L	7 (6.7%)	7 (20.6%)	0.02 *
Dual M + L	10 (9.6%)	10 (29.4%)	<0.005 *
Multi (3+)	14 (13.5%)	11 (32.4%)	0.01 *

^1^ <60 years versus ≥60 years; * statistically significant; C: clarithromycin; A: amoxicillin; M: metronidazole; L: levofloxacin; Multi (3+): resistance to 3 or more antimicrobials.

**Table 6:** Logistic regression analysis of age and antibiotic resistance.

	β_0_ (Intercept)	β_1_ (Age)	*p* Value (LRT)	AUC	*p* Value (AUC)
No Resistance	−1.09	0.00	0.97	0.51	0.82
Clarithromycin	−0.49	0.00	0.75	0.52	0.64
Metronidazole	−0.30	0.01	0.62	0.522	0.66
Levofloxacin	−3.90	0.05	<0.001 *	0.68	<0.005 *
Tetracycline	−2.56	0.01	0.66	0.54	0.58
Amoxicillin	−2.54	0.02	0.18	0.59	0.17
Rifampicin	−1.45	−0.01	0.44	0.54	0.64
Dual C +M	−1.70	0.01	0.23	0.57	0.24
Dual M + A	−2.96	0.02	0.28	0.58	0.32
Dual C + L	−4.54	0.04	0.01 *	0.69	0.02 *
Dual M + L	−4.18	0.05	<0.005 *	0.67	0.01 *
Multi (3+)	−2.89	0.03	0.06	0.61	0.07

LRT: likelihood ratio test; AUC: area under curve; * statistically significant; C: clarithromycin; A: amoxicillin; M: metronidazole; L: levofloxacin; Multi (3+): resistance to 3 or more antimicrobials.

## Data Availability

The original contributions presented in this study are included in the article. Further inquiries can be directed to the corresponding author.

## Abbreviations

The following abbreviations are used in this manuscript:

AST	Antimicrobial susceptibility testing
EUCAST	European Committee on Antimicrobial Susceptibility Testing
*H. pylori*	*Helicobacter pylori*
LRT	Likelihood ratio test
MIC	Minimum inhibitory concentration
PPI	Proton pump inhibitor
SD	Standard deviation
WHO	World Health Organisation
